# An Outline on the Advancements in Surgical Management of Osteoporosis-Associated Fractures

**DOI:** 10.7759/cureus.63226

**Published:** 2024-06-26

**Authors:** Ibrahim A Hakami

**Affiliations:** 1 Department of Orthopedic Surgery, College of Medicine, Shaqra University, Dawadmi, SAU

**Keywords:** bone regeneration, robotic-assisted surgery, kyphoplasty, vertebroplasty, surgical management, osteoporosis

## Abstract

Osteoporosis significantly impairs bone density and increases fracture risk, representing a substantial global health challenge. The effectiveness of traditional treatments such as calcium supplementation and exercise in completely preventing fractures is limited. This review explores recent advancements in surgical techniques and treatment modalities to manage osteoporotic fractures better and improve patient outcomes. Osteoporotic fractures demand specialized surgical techniques due to compromised bone quality. Vertebroplasty and kyphoplasty are minimally invasive procedures that provide rapid pain relief and structural support using bone cement. While vertebroplasty is effective, it carries risks of cement leakage and new fractures. Kyphoplasty, with added balloon inflation, reduces leakage risk and improves vertebral height restoration but is costlier. Cement-augmented screws enhance fixation but can increase adjacent fracture risk and pose long-term complications. Surgical advancements encompass robotic-assisted surgery, offering precision and accelerated recovery, alongside biologic agents like bone morphogenetic proteins (BMPs), which enhance bone healing while reducing secondary interventions and eliminating donor site morbidity. Bone graft substitutes such as calcium phosphate cements enhance biomechanical compatibility, decrease morbidity, and reduce fracture loss and pain. Balloon kyphoplasty aids in height restoration and pain relief and diminishes the risk of subsequent vertebral fractures. Bioglass scaffolds promote bone regeneration by improving bone mineral density and lowering the incidence of new fractures. Optimal perioperative care, including patient selection, nutritional management, and early mobilization strategies, is crucial for mitigating risks in vulnerable populations. While current surgical interventions provide significant pain relief and functional benefits, ongoing research and multidisciplinary collaboration are crucial to prospectively refine these techniques and reduce the burden of osteoporosis. New technologies, such as tissue engineering and gene editing, hold potential for future treatment paradigms.

## Introduction and background

Osteoporosis, a prevalent bone disease characterized by a bone mineral density (BMD) T-score of -2.5 or lower, significantly increases the risk of fractures compared to those with normal bone density [1]. Patients with fragility fractures are at higher risk for further fractures; e.g., patients with a vertebral fracture have a higher risk for another fracture in the subsequent year than those without fractures [2,3]. This condition poses a major health concern globally, affecting approximately 200 million women worldwide [4]. Statistics indicate that one in three women over 50 and one in five men over 50 experience osteoporotic fractures [5]. Chronic inflammatory conditions, where increased cytokines accelerate bone loss; prolonged corticosteroid use, which disrupts bone remodeling; and hyperparathyroidism, where excess parathyroid hormone leads to increased bone resorption, all exacerbate osteoporosis. These factors, along with hormonal changes after menopause-specifically, reduced estrogen levels, significantly accelerate bone loss [6].

Preventive measures, including eating a healthy diet, getting regular exercise, calcium and vitamin D supplementation, and avoiding smoking and excessive alcohol consumption [7], as well as fall prevention strategies, including home hazard assessment and the use of assistive walking devices, are crucial to minimize the risk of fractures [8]. Non-surgical treatments, including the use of medications like bisphosphonates, selective estrogen receptor modulators (SERMs), calcitonin, and parathyroid hormone analogs, are effective in delaying the onset and reducing the severity of osteoporosis manifestations. Hormone replacement therapy (HRT) may also be considered for some postmenopausal women, while newer medications such as denosumab, which is a monoclonal antibody, can help reduce bone resorption [9].

However, in severe cases of osteoporosis, surgery becomes crucial, especially for hip fractures, which carry a high mortality risk [10]. In the U.S. alone, there are over 250,000 hip fractures annually. Vertebral fractures, numbering around 700,000 yearly in the U.S., also often require surgical intervention due to the long-term pain and mobility issues they cause [11]. The incidence of hip fractures in Saudi Arabia signified that the estimated number of hip fractures nationwide in persons older than 50 years in 2015 was about 2,949. This number is projected to increase to about 20,328 in 2050, indicating that nearly seven times as many hip fractures are expected [12].

Osteoporotic bones pose significant challenges during surgical interventions, including issues like screw loosening, refracture, and delayed union or non-union of bones [10]. For instance, the incidence of screw loosening in osteoporotic patients can be as high as 60%, which is markedly greater compared to less than 1-15% in individuals with normal bone density (P < 0.05) [13]. Furthermore, osteoporotic patients face a 50% greater risk of refractures following surgery compared to non-osteoporotic individuals [14]. Delayed or non-union occurs in about 5-10% of osteoporotic fractures, versus 2% in general cases [15]. Additionally, the elderly, who are most commonly affected by osteoporosis, often present with multiple comorbidities, which elevates the risk of surgical complications [16]. This includes a 34.2% increased risk of intraoperative complications, prolonged hospital stays, and higher rates of postoperative infections (P < 0.001), underlining the critical need for specialized surgical protocols and meticulous perioperative management [17,18].

Given these challenges, this review article aims to explore recent advancements in the surgical management of fractures associated with osteoporosis. By examining innovative surgical techniques and new treatment modalities, the review seeks to enhance the understanding of how modern interventions can better support the skeletal health of patients with osteoporosis, ultimately improving their quality of life. This exploration is critical to addressing the growing incidence of osteoporosis and its impact on an aging population.

## Review

Surgical techniques for osteoporotic fractures

Osteoporotic fractures require specialized surgical techniques due to the compromised bone quality. Among the most common are vertebroplasty, kyphoplasty, and the use of cement-augmented screws. These procedures are designed to relieve pain and stabilize fractures, but they have limitations and challenges.

Vertebroplasty

Vertebroplasty, a minimally invasive spinal procedure, involves injecting polymethyl methacrylate (PMMA) bone cement into a fractured vertebral body through the skin to relieve pain and offer structural support [19]. It's highly valued for its rapid pain-relieving effects, with research showing a significant reduction in pain within 24-48 hours after surgery [20]. Studies indicate that more than 90% of patients with osteoporotic vertebral compression fractures (VFs), 70% of those with malignant VFs, and 80% of those with painful hemangioma experience pain relief after vertebroplasty. In one study, there was a significant decrease in pain scores, decreasing from an average of 8.1 out of 10 to 2.6 [21]. In another trial, patients experienced a drop in pain scores from an average of 7.3 on the Visual Analog Scale (VAS) to 5.7 within one week post-procedure [22].

A multicenter randomized trial further validates these findings, reporting sustained benefits for patients undergoing vertebroplasty over 12 months. Alongside rapid pain relief, patients exhibited improved mobility, enhanced physical functioning, and a better quality of life. Moreover, there was a significant decrease in analgesic use, indicating effective long-term pain management. Additionally, vertebroplasty likely contributed to enhanced postural stability and reduced risk of subsequent fractures, which is particularly vital for osteoporosis patients prone to recurrent fractures due to skeletal fragility. The study noted a 42% reduction in Visual Analog Scale (VAS) scores maintained throughout the year post-surgery, compared to a 25% reduction in non-surgically treated patients [23]. Additionally, the procedure's popularity has grown due to its ability to increase quality of life [24]. In a randomized controlled trial, patients who underwent vertebroplasty reported marked improvements in quality-of-life scores measured by the EQ-5D index, with scores improving from 0.62 pre-operation to 0.80 post-operation on a scale where 1 represents full health. These improvements are attributed to pain relief and increased mobility [25].

However, vertebroplasty is not without its risks, primarily associated with the use of PMMA cement. The incidence of cement leakage is reported in approximately 5% to 80% of procedures, depending on the technique used and the condition of the vertebral body [26]. Although most cement leakages are asymptomatic and do not result in clinical complications, about 1% of these cases may lead to serious adverse effects such as nerve root compression or, more rarely, pulmonary embolism [24]. The risk of developing new fractures near the treated vertebrae post-vertebroplasty is another concern, with studies suggesting an increased risk ranging from 12% to 58%. These figures underscore the need for careful patient selection and meticulous surgical technique to minimize potential complications associated with the procedure [27].

Kyphoplasty

Kyphoplasty enhances the method of vertebroplasty by integrating a critical step: the insertion and inflation of a balloon within the vertebral body. This balloon lifts the collapsed bone fragments to restore vertebral height and creates a cavity that is later filled with PMMA to stabilize the fracture [21]. Clinical evidence highlights the procedure’s efficacy, with studies showing that kyphoplasty can be superior in restoring the vertebral body's lost height compared to vertebroplasty [28]. According to data from the Journal of Patient Safety Surgery, patients undergoing kyphoplasty typically report a dramatic decrease in pain, with average scores on a Visual Analog Scale dropping from 7.5 pre-procedure to 2.3 post-procedure [29]. Additionally, functional outcomes measured by the Oswestry Disability Index (ODI) showed significant improvement (p-value 0.0003) post-procedure compared to pre-procedure, underlining significant enhancements in day-to-day activities [30].

The procedure’s benefits may justify its cost through reduced complications: the incidence of cement leakage in kyphoplasty is substantially lower, i.e., 18.4% [31], compared to up to 80% in vertebroplasty [32]. This reduced risk of leakage leads to fewer adverse events, such as nerve damage or pulmonary embolism, which occur in less than 2% of kyphoplasty procedures, potentially reducing the need for further medical interventions and the associated healthcare costs [33]. These factors make kyphoplasty a valuable option for treating severe vertebral compressions, particularly in patients at higher risk of complications from vertebral deformities and subsequent quality of life degradation [34]. Despite its clinical success, kyphoplasty is more costly than simpler procedures like vertebroplasty. The material and equipment costs, including the balloons and high-viscosity cement, contribute to a higher average expense, with average hospital charges approximating $8,100 for kyphoplasty versus $3,319 for vertebroplasty [35]. Infections represent another serious but rare complication. The risk of infection varies but generally remains low, with incidence rates reported as 3.4% depending on the patient's health status, sterility of the procedure, and complexity of the surgery [36].

Cement-Augmented Screw

The cement-augmented screw fixation involves the use of screws that are either pre-coated with or injected with cement at the time of insertion. This technique enhances the anchorage of screws in osteoporotic bone, which is critical for the stability of spinal fixation constructs [37]. Studies have shown that cement augmentation can increase the pull-out strength of pedicle screws by up to 94% compared to non-augmented screws [38]. However, the addition of cement introduces risks of adjacent vertebral fractures due to altered load distribution, nerve damage, vascular injury, and pulmonary embolism, with an estimated increase in the risk of loosening by 22.5%. The use of PMMA cement, while providing immediate structural support, does not integrate with the natural bone, nor does it promote new bone growth [39]. Long-term studies have indicated potential issues with cement, such as cement fatigue and cracking over time, leading to late-onset pain and instability. Additionally, the heat generated during the curing process of PMMA can potentially damage the surrounding bone tissue, reducing its viability and strength [40].

Given the limitations of current surgical options, there is a clear need for advancements that not only stabilize the fracture but also promote bone health and prevent future fractures. Research is ongoing into bioactive cement that not only stabilizes fractures but also supports bone regeneration. These new materials aim to integrate more naturally with the bone, potentially reducing the rate of refracture and improving overall outcomes [41]. Innovative surgical techniques that reduce the invasiveness of procedures and the reliance on non-biological materials like PMMA are also under development [42]. Techniques such as robotic surgery and computer-assisted navigation provide more precise implant placement, potentially reducing the risks associated with traditional methods [43].

Minimal Invasive Procedures

These demonstrate significant advantages in orthopedic surgeries by minimizing tissue damage and reducing periosteal stripping, thus preserving essential blood supply. A study involving 50 patients undergoing fracture repairs highlighted that minimally invasive techniques reduced the duration of hospital stays by an average of 4.3 days and improved early postoperative mobility [44]. In the mini-incision group, both total estimated bleeding (mean 1083.5 ml vs. 1682.3 ml; p < 0.001) and intraoperative bleeding (mean 745.6 ml vs. 1282.8 ml; p < 0.001) were significantly lower compared to the control group. Postoperative blood drainage volume did not differ significantly between the groups (mean 340 ml vs. 399 ml; p = 0.77). However, the mini-incision group had a notably lower rate of requiring allogenic transfusions compared to the control group (8.8% vs. 28.6%; p = 0.02) [45]. Furthermore, minimally invasive surgeries reduce infection and pseudarthrosis risks by conserving the body's biological resources during bone healing, preserving the fracture hematoma, and avoiding periosteal stripping [46].

A study on direct anterior total hip arthroplasty (THA) adheres to minimally invasive principles, emphasizing muscle-sparing techniques over traditional approaches. It aims to reduce soft tissue damage and expedite recovery. Although no significant disparity was observed between short and standard-length stems overall, a correlation emerged suggesting that a higher body mass index (BMI) might result in less damage with a short stem [47]. In a study involving 147 total knee arthroplasties (TKA), it was observed that minimally invasive surgery (MIS) resulted in muscle damage levels similar to those of conventional approaches. However, the severity of muscle damage varied depending on the specific approach used within MIS-TKA [48].

Arthroplasty

Arthroplasty entails surgically replacing a joint with a synthetic material. Total arthroplasty replaces all involved joint surfaces, whereas partial arthroplasty replaces only specific surfaces, leaving others intact. The hip and knee joints are the most commonly replaced [49]. Hemiarthroplasty in intertrochanteric femur fractures among elderly patients may offer advantages over osteosynthesis, yielding high Harris hip scores. Ridha et al. reported that osteosynthesis had a notably higher re-operation rate, with incidences of 40% and 11%, respectively [50]. In another study, combining direct and indirect outcomes revealed a lower rate of revisions (P = 0.000) in patients who underwent total hip arthroplasty compared to those who underwent osteosynthesis, as well as in those treated with hip arthroplasty compared to osteosynthesis (P = 0.000) [51]. However, osteosynthesis can offer advantages to patients who cannot withstand substantial blood loss and prolonged surgical durations. In one study, participants in the osteosynthesis group experienced a mean blood loss of 28 ml, notably less than the 177 ml recorded in the hemiarthroplasty group. Furthermore, osteosynthesis procedures averaged 36 minutes in duration, contrasting with the 57-minute average for hemiarthroplasty. Additionally, osteosynthesis was associated with a lower incidence of blood transfusion requirements. Babcock et al. suggest that hip fractures treated operatively via internal fixation or nonoperatively both carry the risk of non-union. Conversely, fractures managed with arthroplasty are immune to this risk since the fractured proximal portion of the femur is removed to accommodate the arthroplasty replacement [52].

Locking Compression Plate

Locking plates feature screws that thread into the plate, establishing a fixed-angle anchorage for the screws. Although more costly than conventional plates, locking plates offer several benefits. They prevent fragments from being pulled towards the plate and adhere to the eccentric hole principle, thereby eliminating the ability to provide compression over the fracture. Approximately 85% of patients with proximal humerus fractures experience uncomplicated healing, with Constant and Dash's scores reaching normal values. In contrast, reported union rates for distal femoral periprosthetic fractures are around 75% [53]. Unlike conventional plates, which rely on friction at the bone-plate interface, the strength of locking plates stems from the combined stability of all bone-screw interfaces, enhancing overall stability by promoting callus formation. Furthermore, employing long plates optimizes axial stability, while removing screws closest to the fracture site minimizes plastic deformation [54]. In the comparison between locking plate systems (LPS) and non-locking plate systems (N-LPS), LPS demonstrated a stability rate of 83%, which was significantly higher than the 40% stability rate observed in N-LPS. Similarly, the use of LPS resulted in a lower need for maxillomandibular fixation (MMF), with only 63% of cases requiring MMF compared to 93% in N-LPS [55]. Moreover, the transition from extensive to percutaneous approaches minimizes trauma to the periosteum and blood vessels, preserving the blood supply [56].

In a randomized clinical trial comparing the efficacy of 3D locking plates versus standard mini plates, the mean procedure duration for patients treated with 3D locking plates was 49.33 minutes, whereas for those treated with standard mini plates, it was 59.67 minutes. Patients in the standard mini plates group reported significantly higher levels of pain on day one and at one-week post-procedure. However, the incidence of infection was consistent at 6.7% (n = 1) in both treatment groups [57]. In another trial, 79.2% (42/53) patients were able to return to their previous living situations, experiencing manageable pain levels (average 2.4 on the VAS scale) and favorable Iowa pelvic scores (average 85.6) within an average follow-up period of 31.5 months. However, the technique was associated with surgery-related complications in 13% of cases, including wound infections and screw malpositioning. Additionally, 17% of patients experienced postoperative complications, such as urinary tract infections, with one case resulting in pneumonia-related death [58].

While locking plates offer advantages, they necessitate reduction before plate application. If a fracture remains poorly reduced, misalignment may persist post-locking plate application. This is because screws cannot be employed to pull in bone or fragments for reduction.

Advancements in surgical management

Robotic-Assisted Surgery

Robotic-assisted surgery (RAS) revolutionizes minimally invasive procedures through advanced robotic systems. These systems feature robotic arms equipped with surgical instruments, a surgeon-operated console, and a high-definition vision system for a detailed 3D view of the surgical site. Unlike conventional methods like open surgery or laparoscopy, RAS combines the expertise of the surgeon with robotic precision, improving both the quality and safety of procedures. It surpasses traditional fluoroscopy-assisted (FA) techniques, significantly enhancing precision and patient outcomes [59]. A systematic review encompassing 10 articles with 1094 patients highlighted that RAS significantly reduces the rate of cement leakage, a common complication in osteoporotic surgeries, with an odds ratio of 0.27 (95% CI, 0.17-0.42; P < 0.00001) and decreases the number of fluoroscopic exposures by an average of 13.88 times per procedure (95% CI, -18.47 to -9.30; P < 0.00001) [60]. Moreover, patients undergoing robotic-assisted procedures experienced a 77-hour shorter hospital stay (p < 0.001) and reported more substantial early postoperative pain relief, with a decrease in VAS scores of 0.19 points within the first three days (p < 0.001) [59]. These factors are particularly important for osteoporotic patients, who face higher risks of prolonged immobility and pain management issues.

Despite these advantages, the adoption of RAS is hindered by higher initial costs and the need for specialized training. The investment in robotic technology can be considerable, often costing healthcare facilities upwards of $2 million for equipment procurement, with additional ongoing maintenance expenses [61]. However, the long-term savings associated with reduced revision surgeries and decreased length of hospital stays could offset these initial costs [62]. For instance, improvements in Cobb angles immediately post-operation at three days and six months were notably better in the robotic group and significantly improved (P < 0.05), respectively, demonstrating sustained benefits that reduce the need for further interventions. Therefore, while upfront costs are significant, the precision and efficiency of RAS may provide a cost-effective solution for managing osteoporotic vertebral compression fractures (OVCFs) in the long term, especially given the potential to significantly enhance patient outcomes and quality of life.

Biologic Agents

Bone morphogenetic proteins (BMPs) regulate mesenchymal stem cell and cancer stem cell differentiation by binding to Type I and Type II receptors on the cell surface [63]. BMPs, especially BMP2 and BMP7, have revolutionized treating complex fractures and spinal issues, offering significant advantages over traditional bone grafting. They primarily stimulate mesenchymal stem cells (MSCs) and osteoprogenitor cells, driving their differentiation into osteoblasts, which are crucial for bone formation and repair [64]. Following FDA approval for open tibial fractures and anterior lumbar interbody fusion (ALIF), BMP2 has been extensively researched and applied. It notably enhances osteoblastic differentiation and bone matrix deposition, which is particularly beneficial for osteoporotic patients [65]. In a randomized controlled trial involving 450 patients with open tibial fractures, BMP2 at a concentration of 1.5 mg/mL significantly enhanced bone healing and decreased the necessity for secondary interventions compared to standard care, showcasing a substantial improvement in healing rates for osteoporotic bones [66]. Between 2001 and 2006, BMP-7 (OP-1) treated tibial non-unions in 62 Belgian patients, with a 79.6% clinical and 84.9% radiographic healing rate. Median union times were 230 days [64].

However, BMP usage has drawbacks. Arterial stiffness, common in chronic kidney disease (CKD), may be linked to an imbalance in BMP-2 and BMP-7, leading to renal damage, hypertension, and increased pulse wave velocity (PWV) in CKD [67]. It is observed that osteoblast-derived BMP2 is involved in bone quality; thus, deficiency of BMP2 affects bone strength [68]. In cases of unstable thoracolumbar fractures treated with BMP, initially severe bone resorption was observed, although this was followed by effective bone repair over time [68]. These findings underscore the necessity of careful patient selection and dosage optimization to minimize adverse effects. Despite these challenges, the use of BMPs still represents a significant advancement in orthopedic and dental surgeries by reducing the need for secondary surgeries and enhancing the overall bone healing process, making them a valuable tool in the modern surgical repertoire.

Bone Graft Substitutes

Bone graft substitutes, such as calcium phosphate cement, integrate seamlessly into bone tissues without forming a capsule. These resorbable, moldable cement can be injected into bone voids, even in cases of limited surgical access, reducing the need for extensive intervention. It addresses some of the key limitations associated with traditional bone grafting techniques, such as autografts and allografts, and offers a promising alternative by mitigating donor site morbidity and circumventing the limited availability of suitable donor tissue. Calcium phosphate cements are particularly beneficial for osteoporotic patients as they are engineered to mimic the mechanical properties of natural bone, which is essential for maintaining structural integrity and facilitating bone regeneration in compromised bone structures [69].

A meta-analysis including fourteen randomized controlled trials highlights the efficacy of calcium phosphate cements in treating fractures among osteoporotic patients. These studies collectively involved various fracture types, such as distal radial fractures, femoral neck fractures, and tibial plateau fractures. Notably, patients treated with calcium phosphate cement experienced a 68% relative risk reduction in fracture loss compared to those receiving autografts (29% to 86%), while a 56% relative risk reduction in pain at the fracture site when compared with controls (14% to 77%) has been observed [70]. This data underscores the biomechanical compatibility and pain management benefits of calcium phosphate cement over traditional grafting materials in osteoporotic patients. Their application offers a substantial benefit by reducing the need for secondary surgeries and enhancing the overall healing process, making them particularly valuable in the treatment of multimorbid patients or those with diminished regenerative capabilities [71].

Balloon Kyphoplasty

Balloon kyphoplasty (BK) is a significant advance in treating osteoporotic compression fractures of the vertebrae. It's a minimally invasive technique, akin to vertebroplasty, aiming to relieve pain, restore vertebral height, and correct kyphosis. During the procedure, an inflatable bone tamp raises the collapsed vertebral body, restoring height. The balloon is then removed, creating space for bone cement injection at lower pressure and higher viscosity than in vertebroplasty [21]. Balloon kyphoplasty (BK) effectively restores vertebral height and reduces pain compared to standard nonsurgical care. In a meta-analysis comparing BK to percutaneous vertebroplasty (VP) for osteoporotic vertebral compression fractures (OVCF), BK demonstrated significant long-term improvement in kyphosis angle (MD = -2.64, 95% CI = -4.66 to -0.61; p = 0.01) and anterior height of the vertebral body (MD = 3.67, 95% CI = 1.40 to 5.94; p = 0.002), with reduced cement leakage rates (RR = 0.70, 95% CI = 0.52 to 0.95; p = 0.02). However, there were no significant differences in short- or long-term pain scores, disability index scores, operation time, short-term kyphosis angle, or adjacent-level fracture rates [72]. Additionally, BK demonstrates a substantial improvement in SF-36 Pain Catastrophizing Scale (PCS) scores (overall treatment effect 3.24 points, p =.0004) and a notable decrease in back pain (overall treatment effect -1.49 points, p <.0001). Importantly, there was no significant difference observed between groups concerning new vertebral fractures (p =.009) [73]. While the procedure offers numerous benefits, its effectiveness is hindered by the high cost of balloons and cement, as well as the risk of cement leakage, although this risk is lower compared to other minimally invasive techniques [74].

Bioglass Osteoconductive Scaffolds

Bioglass osteoconductive scaffolds are a prospective option for osteoporotic vertebral compression fractures (OVCFs). They have been recently discovered as a viable treatment. Glass-ceramics are a result of partial crystallization of the parent bioactive glass, performed by heating the parent bioactive glass above its crystallization temperature, usually at about 610-630°C. As for glass-ceramics, which are obtained by a sintering process, crystallization and densification of the parent glass, as well as a reduction in porosity and an increase in mechanical strength, occur during the solid structure formation. It is employed to enhance bone regeneration and reduce the risk of recurrent fracture [75]. A study among 116 patients reported being treated with a derivative of bioglass S53P4 (BonAlive®, Bonalive Biomaterials Ltd., Finland). That showed a 90% rapid recovery of bones after one year; 84.5% received only S53P4, while 15.5% received antibiotics alongside S53P4 [76]. The technology not only supports new bone growth but also reduces the incidence of new fractures adjacent to the treated vertebra [77]. The primary drawbacks include the need for precise placement during surgery and potential long-term integration issues, such as scaffold degradation or inflammation [78].

Special considerations for surgical management

Surgical management of high-risk populations, particularly elderly patients with multiple comorbidities, requires meticulous attention to detail and tailored strategies to optimize outcomes. In the elderly, specific surgical considerations are necessitated by diminished BMD and altered physiological responses [10].

Managing patients with multimorbidity in surgical settings involves confronting various challenges due to the presence of multiple comorbid conditions. Firstly, the interaction between these conditions can significantly complicate anesthetic management and surgical intervention. For instance, a patient with diabetes and cardiovascular disease has a higher risk of perioperative complications, such as poor wound healing and cardiac events [79]. Secondly, polypharmacy issues arise as these patients are often on multiple medications, which may interact adversely with surgical drugs or affect the body's response to anesthesia [80]. Additionally, the recovery process is more complex and prolonged because the presence of multiple diseases can impair the body’s natural healing processes and increase the susceptibility to postoperative complications like infections or thromboembolic events. Effective management thus requires a comprehensive and multidisciplinary approach that includes detailed pre-operative assessments, tailored perioperative care plans, and vigilant postoperative monitoring to navigate these complications successfully [81].

Perioperative care for osteoporosis patients encompasses a multidisciplinary approach designed to optimize surgical outcomes through various phases. During the pre-operative phase, thorough assessments are crucial, focusing on patient selection and addressing nutritional deficiencies that are prevalent in osteoporosis patients [82]. Patient selection involves a careful evaluation of the risk-benefit ratio of surgery. Advanced age is a significant consideration, as the prevalence of osteoporosis sharply increases with age. Approximately 200 million people worldwide suffer from the disease, with women over the age of 50 being particularly at risk. For these patients, non-surgical management should always be considered and exhaustively explored before surgical options are pursued. When surgery is deemed necessary, tailored approaches are required. For example, the use of PMMA for screw augmentation in spinal surgeries [19], as well as the utilization of minimally invasive techniques where feasible, can reduce the incidence of postoperative complications, a key concern given osteoporotic fractures [83].

Nutritional deficiencies should be addressed pre-operatively, as about 40%-90% of the adult population suffers from vitamin D deficiency, a critical factor for bone health and recovery. Addressing this through dietary supplements or enriched diet plans can significantly enhance patient readiness for surgery. Additionally, careful surgical planning is implemented to choose the most appropriate, minimally invasive techniques, when possible, which are known to reduce the physical impact of surgery, such as decreased blood loss and shorter hospital stays [84].

Another risk to the osteoporotic elderly cohort includes hardware failure and fractures near the surgical site, with rates of screw loosening in osteoporotic spines reported as high as 10-25% [85]. Surgical techniques adapted to this demographic include the use of larger or longer pedicle screws for enhanced fixation stability and to reduce the risk of hardware failure. Studies suggest that using screws with a larger diameter can increase pull-out strength by up to 30%, which is essential in securing implants in osteoporotic bone [86].

Postoperative care then shifts focus to immediate and long-term recovery processes. Early mobilization is a cornerstone of this phase, aimed at preventing complications associated with prolonged bed rest, such as deep vein thrombosis (DVT), pulmonary embolism (PE), and muscle atrophy [87]. Implementing physical therapy early can significantly improve functional outcomes and reduce the length of hospital stays. For instance, initiating gentle mobility exercises within the first 24 hours post-surgery and progressively increasing activity under supervision can help mitigate the risk of hospital-acquired complications and expedite recovery [88].

Furthermore, a multimodal pain management strategy, including regional anesthesia, can facilitate earlier mobilization and reduce reliance on opioids, which are particularly problematic in the elderly due to increased risks of delirium and respiratory depression [89].

Rehabilitative strategies should emphasize strength training and balance exercises to prevent falls, which are exceedingly common in this group. Statistics show that one-third of the population over 65 experiences falls annually, with a significant number resulting in serious injuries [90]. Thus, a comprehensive approach that integrates meticulous surgical planning, tailored perioperative strategies, and robust rehabilitative care is imperative to ensure the best possible outcomes for elderly patients with multiple comorbidities.

Future directions and challenges

The field of osteoporosis management is on the cusp of transformation, with significant research directed toward advancing surgical techniques and exploring innovative technologies that could revolutionize treatment paradigms. One of the most promising areas is tissue engineering, which aims to develop bone graft substitutes that not only support but actively facilitate the growth and healing of osteoporotic fractures. These substitutes, crafted to mimic the bone's natural extracellular matrix, are becoming increasingly sophisticated, with some showing enhanced integration with existing bone, potentially reducing recovery times [91].

Another exciting frontier is stem cell therapy, particularly the use of mesenchymal stem cells (MSCs), which have shown potential for differentiating into bone cells and regenerating bone tissue. Early trials suggest that MSCs could significantly improve bone density. However, the optimal delivery methods and long-term survival of these cells remain under investigation. Gene editing technologies like Clustered Regularly Interspaced Short Palindromic Repeats (CRISPR) offer a different approach by potentially allowing for the correction of genetic defects that contribute to osteoporosis. Although still in the early stages, experiments have successfully targeted and modified genes in animal models that are crucial for bone density regulation, suggesting a future where genetic predispositions to osteoporosis could be mitigated or even eliminated [92].

The widespread adoption of advanced medical technologies faces challenges due to high costs, necessitating solutions for improved accessibility. Integration into standard practice demands comprehensive training and protocol shifts, potentially met with resistance. Patient acceptance, especially for treatments like gene editing, is hindered by skepticism and misunderstandings, compounded by economic, regulatory, and educational barriers [93].

Preventive measures and early intervention remain pivotal in managing osteoporosis effectively. Current screening practices for osteoporosis need enhancement to identify at-risk individuals sooner, allowing for earlier intervention that could significantly delay or reduce the disease's impact. For instance, widespread calcium and vitamin D supplementation, along with lifestyle modifications, have been shown to decrease fracture rates by up to 30-70%, underscoring the importance of preventive health strategies [74]. Long-term studies are crucial for tailoring treatments, while collaboration among stakeholders is essential to optimize emerging treatments and improve patient care.

## Conclusions

Recent advancements in surgical management offer significant promise for improving outcomes for patients with osteoporosis and osteoporotic fractures. Approaches such as vertebroplasty, kyphoplasty, cement-augmented screws, minimally invasive procedures, robotic surgeries, modern endoprosthesis, and low-contact plates offer rapid pain relief, improved stability, and reduced failure rates. Additionally, they enhance mobility and decrease postoperative complications. Emerging technologies, such as tissue engineering and gene editing, hold promise for further innovation in orthopedic care. Multidisciplinary collaboration is key to overcoming challenges and optimizing surgical interventions, ensuring that patients receive the best possible care. Continued research and investment in preventive measures are essential to further enhance the management of osteoporosis and reduce the burden of this debilitating disease on individuals and healthcare systems worldwide.
